# Chronic stress in solid tumor development: from mechanisms to interventions

**DOI:** 10.1186/s12929-023-00903-9

**Published:** 2023-01-28

**Authors:** Jiajing Yan, Yibing Chen, Minhua Luo, Xinyu Hu, Hongsheng Li, Quentin Liu, Zhengzhi Zou

**Affiliations:** 1 MOE Key Laboratory of Laser Life Science & Guangdong Provincial Key Laboratory of Laser Life Science, College of Biophotonics, South China Normal University, Guangzhou, 510631 China; 2 Department of Gynecology and Obstetrics, First Affiliated Hospital, Genetic and Prenatal Diagnosis Center, Zhengzhou University, Zhengzhou, 450001 China; 3 Department of Breast Surgery, Affiliated Cancer Hospital & Institute of Guangzhou Medical University, Guangzhou, 510095 China; 4 State Key Laboratory of Oncology in South China, Collaborative Innovation Center for Cancer Medicine, Sun Yat-Sen University Cancer Center, Guangzhou, 510631 China; 5 Institute of Cancer Stem Cell, Dalian Medical University, Dalian, 116044 Liaoning China; 6 Guangzhou Key Laboratory of Spectral Analysis and Functional Probes, College of Biophotonics, South China Normal University, Guangzhou, 510631 China

**Keywords:** Chronic stress, Tumor development, Tumor microenvironment, Cancer treatment

## Abstract

Chronic stress results in disturbances of body hormones through the neuroendocrine system. Cancer patients often experience recurrent anxiety and restlessness during disease progression and treatment, which aggravates disease progression and hinders treatment effects. Recent studies have shown that chronic stress-regulated neuroendocrine systems secret hormones to activate many signaling pathways related to tumor development in tumor cells. The activated neuroendocrine system acts not only on tumor cells but also modulates the survival and metabolic changes of surrounding non-cancerous cells. Current clinical evidences also suggest that chronic stress affects the outcome of cancer treatment. However, in clinic, there is lack of effective treatment for chronic stress in cancer patients. In this review, we discuss the main mechanisms by which chronic stress regulates the tumor microenvironment, including functional regulation of tumor cells by stress hormones (stem cell-like properties, metastasis, angiogenesis, DNA damage accumulation, and apoptotic resistance), metabolic reprogramming and immune escape, and peritumor neuromodulation. Based on the current clinical treatment framework for cancer and chronic stress, we also summarize pharmacological and non-pharmacological therapeutic approaches to provide some directions for cancer therapy.

## Background

**Fig. 1:**
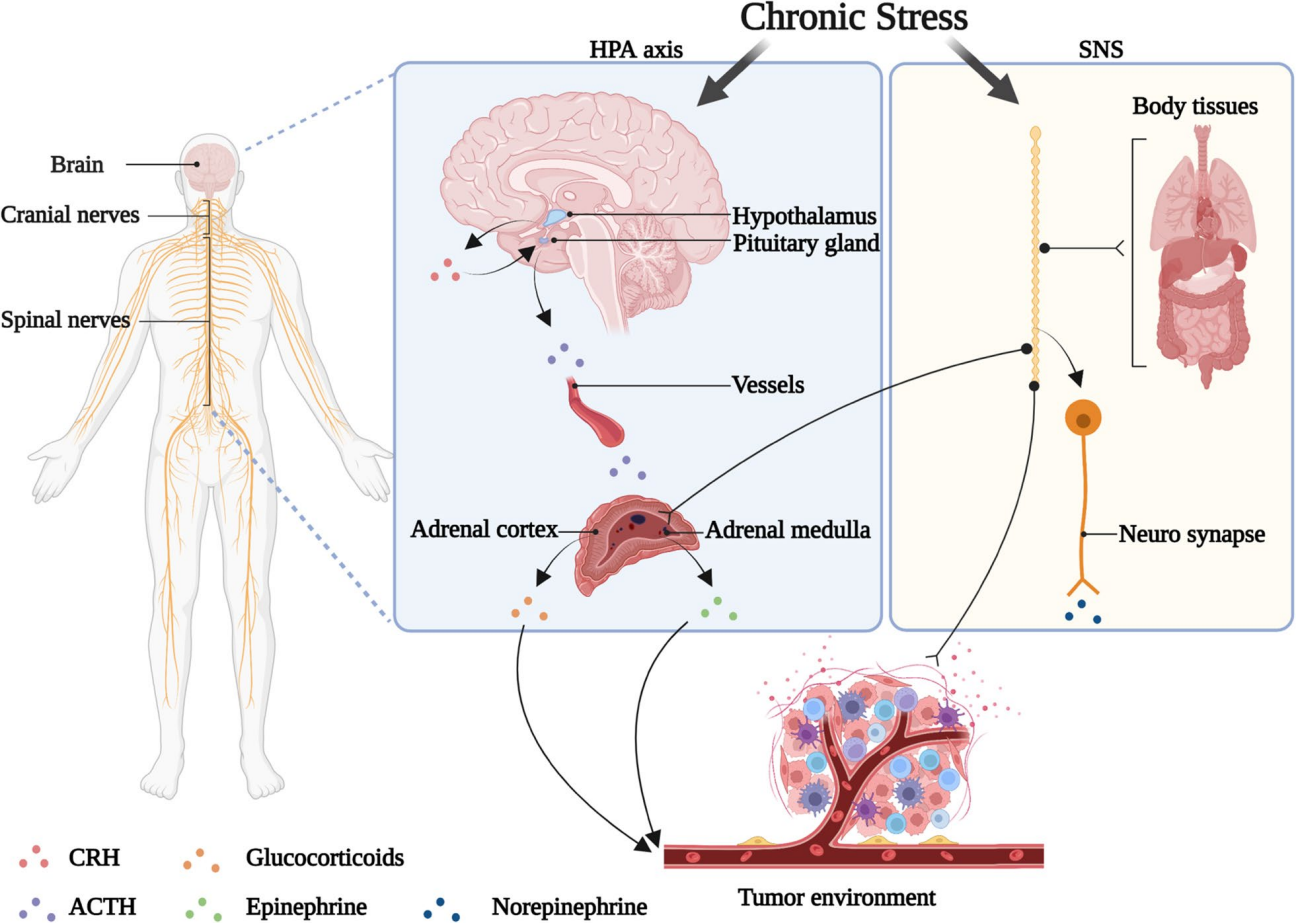
Chronic stress regulates the tumor microenvironment through the neuroendocrine system. The nervous system is composed of cranial and spinal nerves. Under chronic stress, the hypothalamus releases CRH, which triggers ACTH secretion from pituitary to stimulate the secretion of glucocorticoids from the adrenal cortex. Chronic stress also activates the SNS, which directly innervates organs through sympathetic neurotransmitter norepinephrine (NE) release from neuro synapse, promoting the synthesis and secretion of epinephrine (E) from the adrenal medulla. NE generally reaches the TME through nerve fibers, and E/glucocorticoids (GCs) reach it through the blood

Nowadays, the work environment and economic situation of society, especially with the explosion of COVID-19 worldwide, people are under tremendous psychological pressure [1]. Prolonged and repeated exposure to psychological stress results in a range of endocrine and behavioral responses [2]. The neuroendocrine system, which is activated by chronic stress, consists of the hypothalamic–pituitary–adrenal (HPA) axis and the sympathetic nervous system (SNS). Under chronic stress, the hypothalamus releases corticotropin-releasing hormone (CRH). And then CRH triggers the anterior pituitary to secrete adrenocorticotropic hormone (ACTH) to stimulate the secretion of corticosteroids (e.g., glucocorticoids) by adrenal cortex. Chronic stress also activates the SNS, which responds to sympathetic stimulation and cortisol. The excited SNS promotes the release of norepinephrine from nerve fibers and also promotes the synthesis and secretion of epinephrine from the adrenal medulla. Epinephrine, norepinephrine and dopamine are collectively known as catecholamines. HPA and SNS generally regulate the function of nearly all human organ systems through two signaling pathways: one is the release of the sympathetic neurotransmitter norepinephrine via sympathetic nerve terminals to engage adrenergic receptors (ARs) that are expressed on target organs; the other is the release of epinephrine and corticosteroids from the adrenal glands through the bloodstream to reach target organs, which acts on ARs and glucocorticoid receptors (GRs) expressed on target organs (Fig. 1) [3]. Stress hormones (including epinephrine, norepinephrine and glucocorticoids) that reach target organs effectively promote gluconeogenesis and glycogenolysis, raise blood glucose, and improve body metabolism. Glucocorticoids also have the ability to regulate protein metabolism, fat metabolism and water and salt metabolism. Through the above physiological functions, stress-induced hormones increase brain excitability and sensitivity, relax blood vessels, and accelerate heart rate and cardiac conduction velocity.

In acute stress (e.g., acute fight or flight response), elevated cortisol acts via a negative feedback loop and inhibits HPA activity through effects at the pituitary, hypothalamic the paraventricular nucleus and hippocampal levels [2]. However, in chronic stress, cortisol remains elevated, dopamine decreased, negative feedback regulation fails, and cortisol metabolism is reduced, which leads to an increased risk of metabolic syndrome, obesity, cancer, mental health disorders, and cardiovascular disease [2]. In addition, SNS activation does not decay over time and is able to more strongly upregulate norepinephrine levels [1]. Studies in animal models have demonstrated that chronic social stress also increases the growth and distribution of sympathetic nerve fibers in peripheral tissues, thereby upregulating ARs activity in target tissues [4].

Chronic stress affects cancer progression by modulating the tumor microenvironment (TME) through the neuroendocrine system [5]. Recent evidence suggests that epinephrine and norepinephrine and glucocorticoids are major factors in chronic stress-promoted tumor development, which is strongly associated with high malignancy and poor prognosis [6, 7]. Epidemiological studies show that cancer patients have a large burden of mental health disorders and much higher rates of depression and anxiety compared to the general population [8, 9]. Thus, more attention should be paid to the relationship between chronic stress and TME.

In addition to malignant cells, immune and stromal cells (fibroblasts, adipocytes, endothelial cells, etc.) and extracellular components (cytokines, growth factors, hormones, extracellular matrix, etc.) are present in the TME. They surround the tumor cells and are interspersed with the vascular system, lymphatic network, and peripheral nerves [10]. Stress hormones act on the majority of cells in the TME and ultimately promote tumor progression by activating specific receptors associated with many cancer biological processes, including genomic instability, metabolic disorders, proliferation, angiogenesis, metastasis, and immune evasion. One of the main objectives of this review is to summarize the main mechanisms by which chronic stress-associated hormones regulate tumor development, including the interactions between cancer cells and infiltrating immune and stromal cell populations in the TME. Recent studies have shown that tumors can recruit nerves into the TME and form peritumor nerves, which in turn affects tumorigenesis, angiogenesis, invasion and metastasis [11]. This bidirectional interaction between the nervous system and tumors is now becoming a rapidly growing research topic known as cancer neuroscience [12]. This review will also focus on and summarize the currently reported biological mechanisms of chronic stress with the tumor peripheral nerves.

Clinical and preclinical studies have now reported that stress impairs adjuvant and neoadjuvant treatment, including radiotherapy, immunotherapy, and chemotherapy, through modulation by glucocorticoids or catecholamines [13]. However, clinical studies have not adequately addressed this question. In clinical treatment, the anti-tumor immune responses are indispensable in immunotherapy, chemotherapy, and radiation as well, while chronic stress acting on the immune system impairs the efficacy of clinical therapy [14]. Therefore, the synergistic integration of intervene of chronic stress into existing cancer treatment regimens is a major challenge, based on the various treatment options currently available for cancer. Here, we summarized pharmacological and non-pharmacological therapeutic approaches based on the currently available clinical treatment framework to provide some direction for cancer treatment.

## Effect of chronic stress on tumor cells

**Fig. 2:**
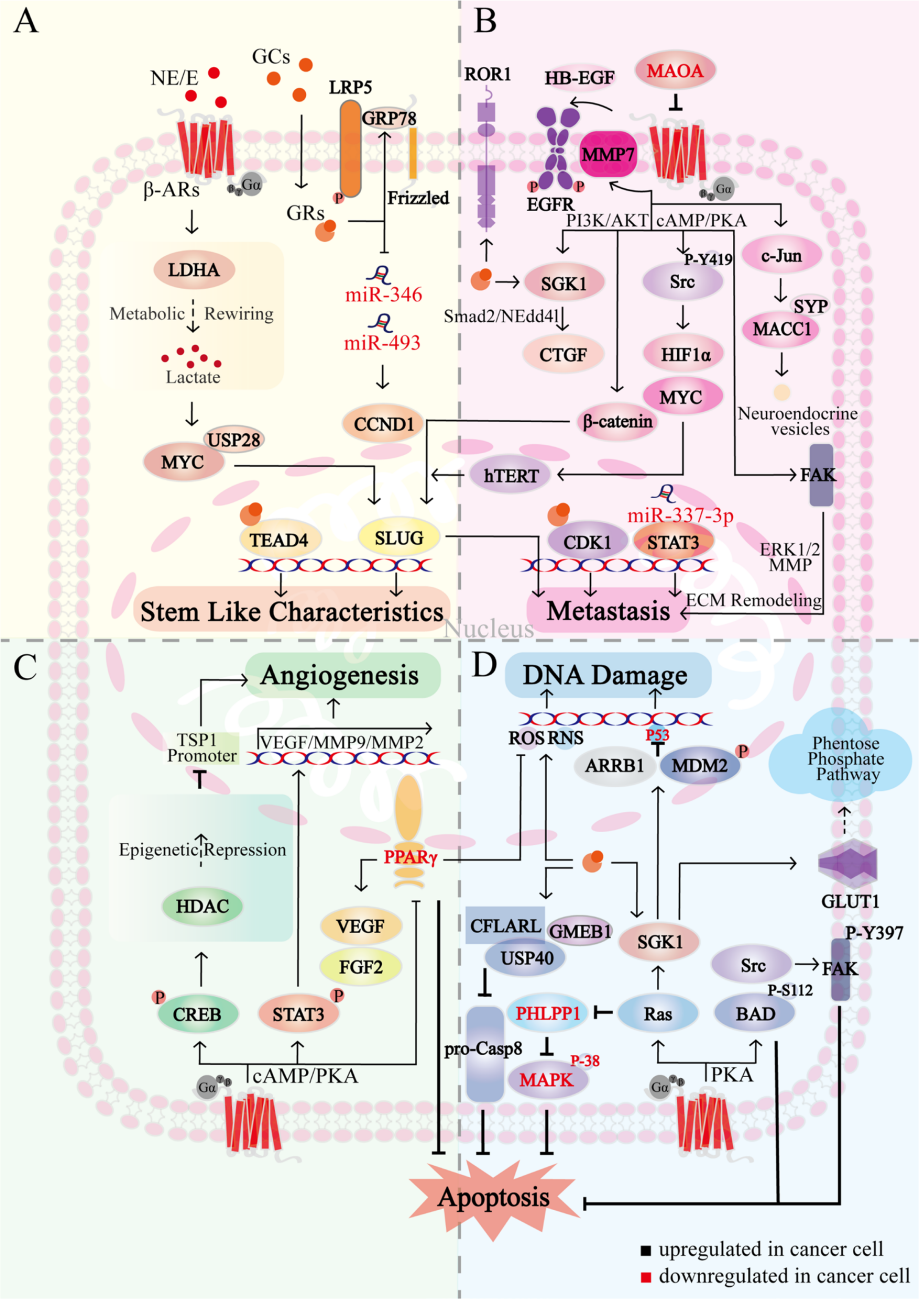
The biological mechanism of chronic stress affecting cancer cells. **A** Chronic stress promotes the stem cell characteristics in cancer cells. Chronic stress-induced E activates LDHA, promotes glycolysis, and leads to lactate secretion, this enhances the interaction between USP28 and MYC, which promotes stem cell-like-associated genes expression via SLUG. Chronic stress-induced GCs promote β-catenin expression through the interaction of GRP78 with LRP5. GCs also downregulate miR-346 and miR-493, which in turn upregulate Cyclin D1 and accelerate the cell cycle. In addition, GCs promote GRs-dependent nuclear accumulation and TEAD4 transcriptional activation, promoting the maintenance of CSCs. **B** Chronic stress accelerates cancer cell metastasis. Chronic stress-induced β-ARs signaling activates FAK via cAMP/PKA, which induces extracellular matrix remodeling via Erk1/2-MMP; Src is also activated by cAMP/PKA, and Y419 phosphorylation of Src amplifies HIF1α and MYC, further inducing hTERT overexpression in the nucleus. hTERT activates SLUG and in turn upregulates metastasis-related genes. β-ARs also promotes β-catenin expression and nuclear localization through PI3K/AKT and increases SLUG promoter activity. NE-induced downregulation of miR-337-3p activates STAT3. β-ARs activates MMP7 and releases HB-EGF to activate EGFR, whereas MAOA can target β2-ARs to reverse these processes. In addition, β-ARs promotes neuroendocrine phenotypic transformation and metastasis through MACC1 upregulation, which binds directly to SYP via c-Jun. GRs contribute to increased ROR1. In addition, GRs increase CTGF expression via PI3K/SGK/Nedd4l-Smad2 and promotes lung metastasis. GRs localize to the CDK1 promoter in nucleus and stimulate CDK1 through epigenetic regulation. **C** Chronic stress promotes angiogenesis. β-ARs activates CREB and STAT3 via cAMP/PKA, STAT3 translocates to the nucleus and stimulates VEGF and MMP2/9 transcription, CREB targets HDAC2 activation, which epistemically represses TSP1. In addition, PPARγ inhibits VEGF by suppressing ROS, while β-ARs signaling reverses the effect of PPARγ on VEGF/FGF2. **D** Chronic stress promotes DNA damage accumulation and anti-apoptosis. β-ARs mediates Src/FAK and BAD anti-apoptotic pathways by PKA, BAD-S112 phosphorylation and FAK-Y397 phosphorylation gain resistance to apoptosis. GCs stabilize CFLARL through GMEB1-USP40 interaction, inhibit pro-CASP8 activation, thereby inhibiting apoptosis. SGK1, a key downstream effector of Ras, promotes glucose-mediated carbon flux into multiple metabolic pathways to inhibit oxidation by GLUT1 activation; in addition, Ras blocks apoptosis by reducing PHLPP1, which activates p38 MAPK to promote apoptosis. Elevated NE/E and GCs increase MDM2 activity and decrease p53 function via SGK1 and ARRB1, β-ARs and GRs also stimulate the production of ROS and RNS, ultimately leading to DNA damage accumulation

Chronic stress-induced catecholamines and glucocorticoids directly act on cancer cells and promote tumor development [5]. Stress hormones bind to the corresponding intracellular receptors to promote inflammation, angiogenesis, genomic instability, metastasis, and stem cell-like related genes expression through change of epigenetics or activation of multiple pathways in tumor cells (Fig. 2) [15]. Not only that, chronic stress-induced tumor cells acquire apoptosis resistance and resist cancer therapy [16]. We herein discuss the functional effects of chronic stress on tumor cells and the pathways that impair cancer therapy.

### Stem cell-like characteristics

Cancer stem cells (CSCs) have a high self-renewal capacity and are responsible for cancer recurrence and resistance to radiotherapy and chemotherapy [17]. Psychological stress promotes stem-like characteristics and tumorigenic potential in cancer cells, including a significant increase in self-renewal genes expressions, such as *CTNNB*, *OCT4*, and *NANOG* [18, 19]. Chronic stress-induced epinephrine enhances lactate dehydrogenase A (LDHA)-dependent glycolysis, which leads to increased lactate secretion. Excess lactate promotes ubiquitin-specific protease 28 (USP28) to stabilize MYC proteins, which transcriptionally active *SLUG* expression to enhance breast cancer stem cell-like features (Fig. 2A) [18]. Chronic stress-induced neurotransmitter also activates CSCs through multiple cAMP-mediated pathways (including ERK, AKT, CREB, SHH, and ALDH-1) in non-small cell lung cancer (NSCLC) [20]. Zheng et al*.* demonstrated that stress-induced excess cortisol increases stem cell properties by enhancing the endoplasmic reticulum stress protein GRP78 (Fig. 2A) [21]. Glucocorticoids have been reported to down-regulate miR-346 and miR-493 levels, which in turn upregulate Cyclin D1 expression and accelerate the cell cycle (Fig. 2A) [22]. In addition, glucocorticoids promote breast cancer cell resistance to cytotoxic compounds like taxanes during cancer treatment through the interaction between GRs and TEA domain transcription factor 4 (TEAD4) [23]. These findings suggest that chronic stress-induced hormones upregulate the expression of proliferation-related genes through multiple pathways, thereby enhancing the stem-like properties of cancer cells.

### Metastasis

Cancer metastasis consists of at least two rate-limiting steps, the entry of metastatic tumors into the systemic circulation and the colonization of circulating tumor cells in distant organs [24]. Chronic stress is involved in key steps of tumor metastasis, endows cancer cells with mesenchymal-like characteristics to enhance their capacity of metastasis and invasion [25]. Numerous key molecular switches activated by ARs have been identified to regulate metastasis in solid tumor. In human ovarian cancer samples, norepinephrine correlates strongly with pSrc Y419 and pSrc S17 [26]. Src as a cytoplasmic tyrosine kinase further amplifies the signaling cascade of HIF-1α and c-Myc to induce human telomerase reverse transcriptase (hTERT) overexpression. Subsequently, hTERT induces *SLUG* expression, upregulates various metastasis-associated genes [27]. β-ARs also promote β-catenin expression and nuclear localization via PI3K/AKT, increasing *SLUG* promoter activity, thereby promoting epithelial-mesenchymal transformation and invasion of ovarian cancer cells (Fig. 2B) [28]. Additionally, norepinephrine-induced downregulation of miR-337-3p further activates STAT3 for lung metastasis of breast cancer (Fig. 2B) [29]. Focal adhesion kinase (FAK) is increased in prostate cancer patients with metastatic features and high depression scores. Activation of FAK is dependent on the cAMP/PKA signaling pathway and regulates extracellular matrix remodeling via matrix metalloproteinase (MMP) release to promote tumor invasion (Fig. 2B) [30]. Monoamine oxidase A (MAOA), a catecholamine neurotransmitter-degrading enzyme, inhibits metastasis by suppressing β-ARs and ARs-mediated epidermal growth factor receptor (EGFR) signaling (Fig. 2B). MAOA is significantly downregulated in clinical hepatic carcinoma samples [31]. Catecholamines upregulate metastasis‐associated in colon cancer 1 (MACC1), which binds directly to synaptophysin (SYP) via the β2-AR/c-Jun signaling pathway, thereby promoting the neuroendocrine phenotypic transformation of gastric cancer cells and accelerating metastasis (Fig. 2B) [32].

A critical step in metastasis also includes the formation of distant metastatic niches. Studies have shown that adrenergic stimulation upregulates C-C motif chemokine ligand 2 (CCL2) of lung stromal cells and C-C motif chemokine receptor 2 (CCR2) of monocytes, which results in macrophage recruitment and infiltration into the pre-metastatic lung and promote lung metastatic colonization of circulating tumor cells [33, 34]. Obradovic et al. demonstrate that glucocorticoids upregulate the receptor-tyrosine-kinase-like orphan receptor (ROR1), which mediates the lung metastatic colonization process, thus reducing patient survival (Fig. 2B) [35].

Notably, glucocorticoids such as dexamethasone (Dex) have been widely used as combination drugs in cancer treatment to combat chemotherapy-induced side effects (e.g. nausea, edema, allergic reactions) [36]. Dex directly inhibits platinum-based chemotherapy-induced 5-hydroxytryptamine 3 (5-HT^3^) receptors, which mediate the physiological and pathological process of vomiting [37]. Dex also relieves pain in cancer patients by inhibiting the synthesis and release of prostaglandins [38]. However, Dex promotes breast cancer metastasis in the standard hypersensitivity reaction to paclitaxel regimen, which should raise concerns during the treatment of clinical breast cancer. Mechanistically, Dex links with GRs on tumor cells to upregulate serum glucocorticoid-induced kinase 1 (SGK1) expression, which in turn mediates cancer metastasis through connective tissue growth factor (CTGF) (Fig. 2B) [39]. Dex also promotes proliferation and invasion of colon adenocarcinoma by stimulating *CDK1* gene expression (Fig. 2B) [40]. Based on these findings, it can be concluded that the effects of Dex are double edged, and the optimal choice is to modify paclitaxel formulations to reduce Dex application.

Given the psychological reactions and physiological stress responses of cancer patients in the perioperative period, surgical excision of primary solid tumors is often accompanied by the development of residual malignant cells metastases [41]. Depression/anxiety is associated with recurrence in breast cancer patients, with recent animal studies providing supportive evidence [42]. Chronic stress impairs TLR-9 immunostimulant (CpG-C) to block cancer metastasis efficacy in the CT-26 metastasis model and the melanoma spontaneous metastasis model. Only the combination of CpG-C and GR and β-AR blockers improve long-term recurrence-free survival after resection of primary metastatic tumors in mice [43].

### Angiogenesis

Angiogenesis is a complex process, which usually depends on the balance between activators and inhibitors of angiogenesis [44]. Normal angiogenesis is essential for the development and growth of tissues, but pathological angiogenesis contributes to the spread and growth of cancer by providing nutrients and oxygen [45]. Thaker et al*.* show that vascularization is markedly increased in stressed animals and is accompanied by upregulation of MMP-2, MMP-9, and vascular endothelial growth factor (VEGF) [46]. This is involved in β-ARs/cAMP/PKA/STAT3 signaling, where activated STAT3 is translocated to the nucleus to bind to specific DNA sites, then stimulates transcription of angiogenesis-related genes (Fig. 2C) [47]. After CREB activation by β-AR, histone deacetylase 2 (HDAC2) epigenetically inhibits thrombospondin-1 (TSP1), a potent angiogenesis inhibitor, thereby promoting angiogenesis and prostate cancer progression (Fig. 2C) [48]. Recent studies have shown that peroxisome proliferator-activated receptor γ (PPARγ) and its agonists induce apoptosis by inhibiting angiogenesis, whereas β2-AR activation reverses this process (Fig. 2C) [49]. Furthermore, in colorectal cancer and melanoma mice, chronic stress impairs the effect of sunitinib, an inhibitor of multiple tyrosine kinase receptors with antiangiogenic and antitumor activity [50].

### Cell death and DNA damage

The pathways of cell death are conserved, clinical cancer therapy has aimed to eliminate cancer cells as effectively as possible through programmed cell death or apoptosis. The primary mechanism of radiation therapy is direct damage to tumor cells in the radiation field. The adrenergic response directly increased tumor cell resistance to radiation in vitro and blocked radiation-induced apoptosis [51, 52]. However, it remains to be elucidated how stress affects the radiation resistance of tumor cells and blocks apoptosis.

Chemotherapy kills cancer cells with chemical drugs, blocks DNA synthesis and induces apoptosis. However, in vitro and in xenograft mouse models, chronic stress hormones impair the efficacy of chemotherapy with cisplatin, paclitaxel, and TRAIL in pancreatic cancer [16]. BCL2-associated death promoter (BAD) phosphorylation is necessary to inhibit apoptosis, and epinephrine mediates the β2-AR/PKA/BAD anti-apoptotic signaling pathway, thereby reducing treatment sensitivity and accelerating cancer progression (Fig. 2D) [53]. β2-AR/Src signaling axis not only regulates tumor metastasis but also gains resistance to apoptosis through phosphorylation of FAK Y397 (Fig. 2D) [54]. In addition, β2-AR and human epidermal growth factor receptor-2 (HER2) constitute a positive feedback loop in human breast cancer cells that mediates resistance to trastuzumab-targeted HER2 therapy [55]. In contrast, β2-AR antagonists significantly downregulate Cyclin D1 expression and inhibit the formation of Cyclin D1/CDK4/6 complexes, resulting in G1/S phase arrest [56, 57]. Like epinephrine, stress-induced glucocorticoids enhance cancer cells resistance and reduce the efficacy of chemotherapy agents (such as paclitaxel, doxorubicin, TRAIL, and 5-fluorouracil) by anti-apoptosis mechanisms [58–60]. An et al*.* find that glucocorticoid modulatory element-binding protein 1 (GMEB1) interacts with the deubiquitinase USP40 to stabilize CFLAR_L_ and inhibit pro-caspase 8 activation, thereby inhibiting apoptosis in NSCLC cells (Fig. 2D) [61]. While the GRs blocker mifepristone can increase paclitaxel efficacy in triple-negative breast cancer [62].

Anti-anoikis is a marker of malignant transformation. In addition to increasing cancer cell survival in the absence of stromal attachment, anti-anoikis promotes migration, re-attachment, and secondary sites colonization [63]. Overexpression of oncogenes such as Ras, Raf, and Src and tumor suppressor genes downregulation such as phosphatase and tensin homolog and p53 help prevent anoikis [64]. For example, as a key downstream effector of Ras, SGK1 increases glucose uptake via glucose transporter protein 1 (GLUT1), which promotes glucose-mediated carbon flux into multiple metabolic pathways to inhibit anoikis (Fig. 2D) [65, 66]. Ras also reduce the phosphatase PHLPP1 expression, which promotes anoikis by activating p38 MAPK (Fig. 2D) [65].

A hallmark of cancer is genomic instability, which is linked to a greater tendency to DNA damage accumulation. In response to stress signals, the tumor suppressor p53 transcriptionally regulates target genes to initiate apoptosis and cell cycle arrest [67]. Chronic stress elevated glucocorticoids and catecholamines increase MDM2 activity and decreases p53 function by SGK1 [68] and Gs-protein-dependent-PKA/β-arrestin-1(ARRB1) pathways (Fig. 2D) [69], thereby synergistically leading to the DNA damage accumulation. In addition, exposure to cortisol and NE significantly increases the levels of reactive oxygen species (ROS) and reactive nitrogen species (RNS) that interact with DNA, also causing extensive DNA damage (Fig. 2D) [70]. Stress-induced G1 phase arrest significantly reduces the efficacy of paclitaxel in triple-negative breast cancer [71]. Several studies have addressed the effects of chronic stress on DNA integrity, but the specific cellular mechanisms underlying the effects on genome stability remain poorly understood. Moreover, it has not been elucidated whether adrenergic inhibitors of DNA damage repair sufficiently increase spontaneous tumorigenesis in vivo.

It is certain that stress hormones simultaneously influence the various biological mechanisms that promote cancer progression. Some key molecular switches, such as Src and Ras, which regulate important protein families such as MYC and FAK, enhance tumor cells' anti-apoptotic capacity to combat tumor suppression response and cancer therapy in the organism. At the same time, these molecular switches promote tumor cell stemness development and metastasis-related gene expression through transcription factors such as SLUG and β-catenin, accelerating the infiltration of blood vessels and lymphatic vessels around the TME and preparing the tumor for distant metastasis. Intervention in these critical molecular switches may provide new avenues for clinical cancer therapy. It is important that future work should explore additional key molecular switches linking chronic stress to these networks of biological mechanisms.

## Effects of chronic stress on immune cells

A growing number of studies suggest that chronic stress can promote an inflammatory TME to activate the interaction between cancer cells and inflammatory immune cells [72]. Immune cells undergo metabolic changes after migrating to TME, including tumor-associated macrophages (TAMs), dendritic cells (DCs), myeloid-derived suppressor cells (MDSCs), and tumor-infiltrating lymphocytes (TILs) [73]. To control tumor growth, various cells communicate through direct contact or producing cytokines and chemokines and act in autocrine and paracrine ways [74].

Stress also modulates immune cell function by affecting tissue blood flow. Chemogenetic activation of the SNS or treatment with adrenergic receptor agonists induces vasoconstriction and reduced local blood flow [75]. This leads to sudden hypoxia in the TME, which triggers rapid calcium signaling in leukocytes, impairs leukocyte migration, and promotes tumor progression [76]. SNS regulating tissue hypoxia may have an impact on antitumor immunity, leading to transient suppression of CD8^+^ and CD4^+^ T cell motility in tissues [77].

### Natural killer (NK) cells

**Fig. 3:**
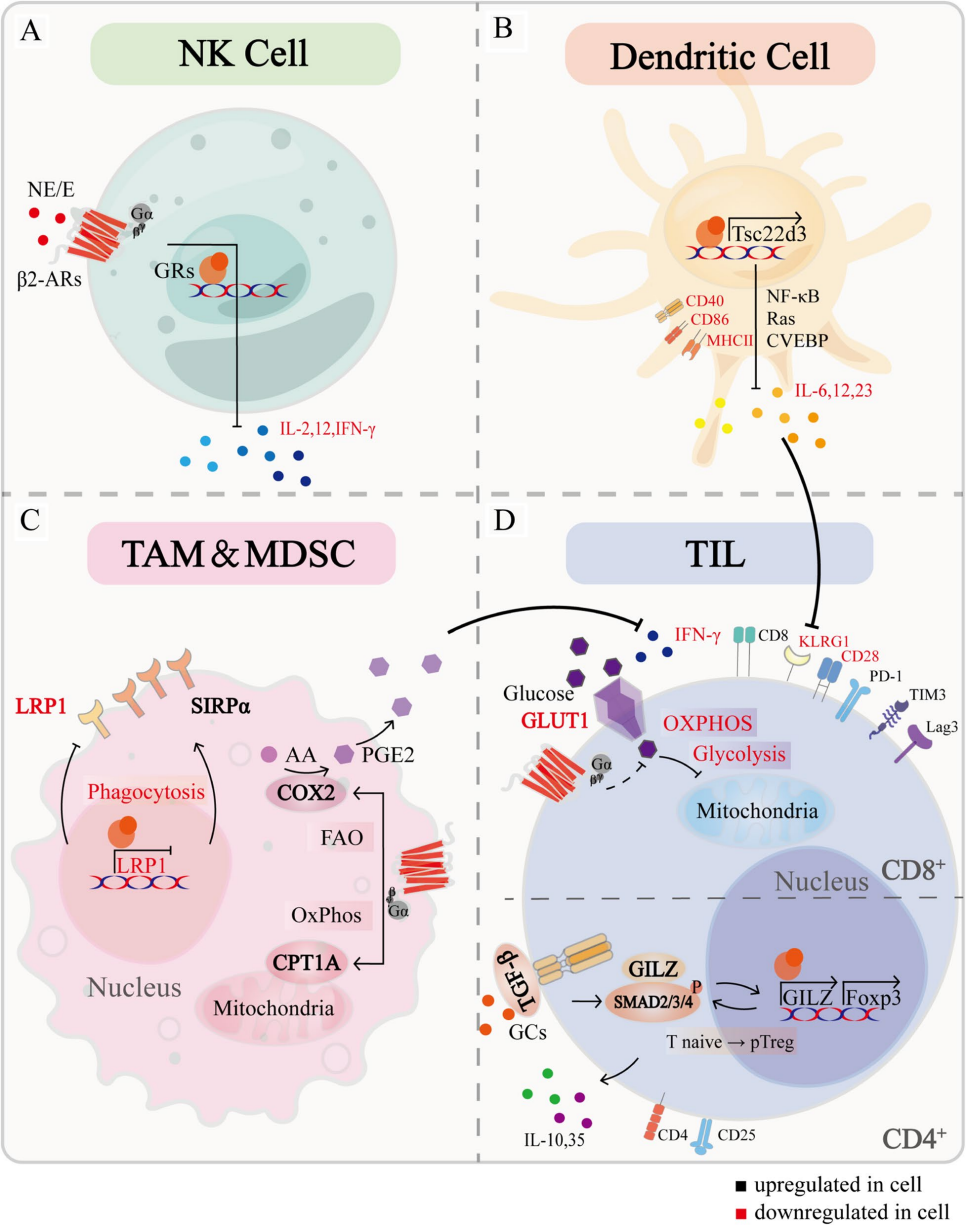
The biological mechanism of chronic stress affecting immune cells. **A** NK cell: E/NE inhibits cytokines production, including IL-12, IFN-γ and IL-2, through DNA-dependent mechanisms activation and protein synthesis. **B** Dendritic cell: chronic stress downregulates maturation-related markers such as CD40, CD68, and MHC II receptors in DCs. GRs upregulate TSC22D3 expression, mediate immunosuppressive effects through NF-κB, Ras and CVEBP, reduce pro-inflammatory cytokines IL-6, 12, and 23 productions, and block antigen presentation, resulting in the inability of TILs to acquire mature phenotype, as evidenced by low KLRG-1. **C** TAM & MDSC: chronic stress mediates metabolic reprogramming of macrophages and promotes immunosuppression. β2-ARs increases oxidative phosphorylation and promotes CPT1A expression; β2-ARs also increases FAO, which increases PGE2 production through COX2 overexpression and inhibits IFN-γ production by CD8^+^ T cells. GCs-GRs inhibit LRP1 and increase SIRPα, leading to an imbalance in the LRP1/SIRPα axis and inhibiting the phagocytosis of tumor cells by macrophages. **D** TIL: chronic stress reshaped the TIL phenotype in the TME, CD4+ TIL exhibit PD-1^+^, FOXP3^+^, CD8^+^ TIL exhibit PD-1^+^, TIM3^+^, Lag3^+^, IFN-γ^−^, CD28^−^. β2-ARs decreased GLUT1 and glucose uptake, resulting in reduced glycolysis and oxidative phosphorylation. GCs-GRs induce GILZ expression, which synergistically induces FoxP3 expression with TGF-β and SMAD2/3/4, further enhancing TGF-β signaling and promoting the conversion of naive T cells to Treg cells. Treg cells can produce immunosuppressive cytokines, including IL-10 and IL-35

NK cells are innately cytotoxic cells that play an important role in targeting early and effective immune responses to infection and cancer [78]. NK cell activity suppression in the perioperative period is due to excessive catecholamine secretion, this process is related to immune responses [79]. Epinephrine/norepinephrine inhibits cytotoxicity and cytokine production (including IL-2, IFN-γ, and IL-12) in NK cells by activating DNA-dependent mechanisms and protein synthesis (Fig. 3A) [80, 81]. Compared to catecholamines, glucocorticoids are considered to be secondary mediators of stress-induced NK cell cytotoxicity inhibition [82].

### Dendritic cells (DCs)

Chronic stress impairs DCs maturation, which is involved in the downregulation of maturation-related factors, such as CD40, CD68, and MHC II receptors, thus reducing antigen presentation and pro-inflammatory cytokines production (including IL-6, IL-12, and IL-23) [83]. Chronic stress also impairs the ability of activated DCs to migrate from the periphery to draining lymph nodes, which directly impairs the initiation of CD8^+^ T cell responses in vivo [84]. The specific mechanism is that stress-induced glucocorticoid-inducible factor TSC22D3 mediates immunosuppressive effects through homo- and heterodimerization with Ras, NF-κB, and CCAAT enhancer-binding protein (CVEBP), thereby blocking type I interferon response and IFN-γ^+^ T cell activation in DCs (Fig. 3B) [7]. In mouse melanoma model, the aforementioned functional alterations of DCs prevents cytotoxic T lymphocytes from acquiring a fully mature effector phenotype in vivo, manifested as low expression of KLRG-1 (Fig. 3B) [84]. PLGA-MS vaccination induces prophylactic and therapeutic protection against aggressive melanomas and promotes CD8^+^ T cell production [85]. However, the reduced tumor protection against immunization in stressed mice after vaccination may suggest that it is important to sufficiently understand the effects of chronic stress on DC function in the therapeutic environment [84].

### Tumor-infiltrating lymphocytes (TILs)

T cells are widely classified according to the central markers (CD8 and CD4) and their receptor subunits [86]. T cell receptor-induced activation drives metabolic reprogramming of T cells (including increased glycolysis and oxidative phosphorylation) to meet essential biosynthetic requirements, this process is remodeled by chronic stress. Studies have shown that adrenergic signaling decrease GLUT1 expression in CD8^+^ T cells, leading to decreased glucose uptake and reduced glycolysis. Not only that, β-ARs-mediated mitochondrial respiration inhibition cause CD8^+^ T-cell mitochondrial dysfunction during activation (Fig. 3D) [87]. Blocking β-ARs signaling increases the metabolic reprogramming in TILs. This is related to increased costimulatory molecule CD28 and antitumor functions, including IFN-γ production, the proliferation of antigen-specific CD8^+^ T cells, and cytolytic killing capacity [84, 88]. Chronic stress also contributes to T-cell exhaustion. One possible mechanism is that β-ARs regulate immune checkpoint expression, which strongly suppress anti-tumor immune responses, characterized by increased PD-1, TIM-3, and Lag3 expression [89]. In breast cancer patient samples, the immune checkpoint molecules expression is positively correlated with sympathetic nerve density, and their expression levels correlated with the recurrence rate of breast cancer patients [90]. Blocking β-ARs signaling results in checkpoint genes expression decreased in CD8^+^ TILs, such as *Ceacam1*, *CD160*, *Btla*, *CD274*, and *Tigit* [89].

Immunotherapy aims to enhance the anti-tumor CD8^+^ T cell response. The most widely used immunotherapy is monoclonal antibodies designed against immune checkpoint molecules, such as PD-1, CTLA4, and 4-1BB, or other stimulatory molecules [91]. However, the effect of chronic stress hormones on the aforementioned T cells leads to the failure of immunotherapy targeting T cells [84, 88, 92]. Notably, positive environmental stimulation (eustress) contributes to reshaping the immunosuppressive microenvironment, enhancing CD8^+^ T cells antitumor immunity and overcoming resistance to therapeutic PD-L1/PD-1 blockade [93]. The use of corticosteroids is corrected with poorer prognosis in patients with NSCLC, which reduce efficacy of immune checkpoint inhibitor therapy [94, 95]. However, the exact mechanism of how glucocorticoids affect immunotherapy remains elusive.

CD4^+^ helper T cells play a role in antitumor immunity response by inducing CD40 ligand expression to stimulate CD40 on DCs to promote CD8^+^ T cell initiation [96]. CD4^+^ T cells differentiate based on various cytokines [97]. It has been shown that β2-AR activation of CD4^+^ T lymphocytes inhibits Th1-cytokine production and cell proliferation [98]. Interestingly, CD4^+^ T cell naive and Th1 cells express the β2-AR, while the Th2 cells do not, and the result suggests that β-ARs regulate the CD4^+^ T cells' Th1/Th2 differentiation, polarizing them toward the Th2 phenotype [99]. Treg cells are usually described as tumor-promoting CD4^+^ CD25^+^ FoxP3^+^ T cells, which produce immunosuppressive cytokines, such as IL-35, IL-10, and TGF-β [100]. Glucocorticoid-induced leucine zipper (GILZ) promotes Treg cells production, which enhances TGF-β signaling by binding to Smad2/3/4 and promoting FoxP3 expression (Fig. 3D) [101]. The balance between Th17 and Treg cells has emerged as an essential factor in the modulation of autoimmunity and cancer [97]. However, the balance of Th17 and Treg cells in tumor progression in the context of chronic stress remains unreported.

### TAMs and MDSCs

The macrophages residing within TME are known as TAMs, which are the main infiltrating immune cells in TME. Psychological depression promotes intratumoral infiltration of TAMs and is correlated with poor clinical prognosis and resistance to tumor therapy due to the immune-suppressive and tumor-promoting activity of TAMs [102, 103]. Catecholamines similarly increase the recruitment of MDSCs, which are immature bone marrow mononuclear cells characterized by a CD11b^+^ Gr1^+^ phenotype [104]. Chronic stress promotes CXCL2/CXCL3 secretion of tumor cells, induces CXCR2 expression of myeloid cells, and thus facilitating spleen myeloid cells movement into tumor tissue through the CXCL2/CXCL3-CXCR2 axis [105]. Besides, enhanced adrenergic signaling mobilizes tumor cells to secrete monocyte/macrophage chemotactic factor CCL2, which increases CD14^+^/CD68^+^ macrophage infiltration in the TME [106]. Glucocorticoids-GRs signal inhibits low-density lipoprotein receptor-related protein-1 (LRP1) expression in tumor-associated macrophages, and increases signal regulatory protein alpha (SIRPα) expression in macrophages, leading to an imbalance in the LRP1/SIRPα axis, which inhibits the phagocytosis of tumor cells by macrophages (Fig. 3C) [107]. Repeated social defeats result in the recruitment of bone marrow-derived monocytes to the brain, where they increase neuroinflammation, leading to prolonged anxiety-like behavior [108], and forming a vicious circle.

Functionally, chronic stress mediates the metabolic reprogramming of macrophages and promotes immunosuppression [109, 110]. β2-ARs signaling in MDSCs or TAMs reduces glycolysis and increases oxidative phosphorylation and fatty acid oxidation (FAO). The latter two are critical metabolic pathways for driving the immune-suppressive function of bone marrow cells [111], and increased FAO promotes prostaglandin E2 (PGE2) production through cytochrome c oxidase subunit 2 (COX2) overexpression (Fig. 3C) [110]. PGE2 is a critical lipid metabolite that effectively recruits neutrophils and macrophages, strongly suppresses both the proliferation and IFN-γ production of CD8^+^ T cells (Fig. 3C) [112]. Ben-Shaanan et al*.* show that positive emotions reduce norepinephrine levels in bone marrow, decrease MDSCs production, and reduce the suppressive effects of MDSCs on T cell proliferation and effector phenotypes in tumor-bearing mice [113].

On the other hand, chronic stress-induced catecholamines promote macrophage M2 polarization [114]. M2 macrophages are commonly associated with tumor metastasis and angiogenesis, as evidenced by increased expression of pro-metastatic genes, production of pro-angiogenic molecules and promotion of perivascular extracellular matrix degradation [115]. TAMs signal to tumor cells via COX2/PGE2 in response to β-ARs signaling to generate VEGFC required for lymphatic remodeling [116]. VEGFC is central to lymph angiogenesis, which increases lymphatic tumor cell dissemination, leading to increased lymph node metastasis [117].

It is reasonable that metabolic reprogramming and polarization of macrophages by chronic stress play a key role in tumor development. Understanding the mechanisms by which chronic stress drives their immunosuppressive functions could help improve cancer immunotherapy and contribute to the discovery of new therapeutic approaches.

These data suggest that chronic stress reshapes immune cells in multiple ways, thereby promoting tumor progression. Stress directly inhibits the anti-tumor function of TILs and NK cells through epigenetic or metabolic reprogramming, which also leads to immunotherapy failure. Stress hormones also indirectly alter the immune function of macrophages and DC cells, leaving TILs unsupported and ultimately leading to uncontrolled tumor cell proliferation capacity.

## Effects of chronic stress on tumor-associated stromal cells

Cancer-associated fibroblasts (CAFs) are the largest stromal cell population in the TME and play an active role in shaping TME to support tumor cell survival, metastasis, angiogenesis, immunosuppression, and treatment resistance [118]. Nagaraja et al. have shown that epinephrine stimulates tumor cells to produce inhibin-βA, which drives elevated levels of collagen production by CAFs, such as COL3A1, COL5A1, COL5A2, and COL11A1 [119]. Increased collagen expression is associated with cancer progression and poor prognosis in breast cancer patients. Collagen accumulation enhances the stemness of cancer cells, induces apoptosis resistance, and promotes cancer metastasis [120].

Normal adipocytes around the tumor are driven into cancer-associated adipocytes (CAAs) by cancer cells. Cancer cells capture metabolites from stromal adipocytes via CAAs and become metabolic parasites [121, 122]. Avril et al*.* have shown that adrenergic stimulation changes the secretome of CAAs, subsequently promoting cancer cell proliferation [123].

In addition to CAFs and CAAs, endothelial cells are also the main targets of adrenergic nerves in the TME [124]. β-ARs signaling promotes angiogenesis by altering aerobic glycolysis in endothelial cells [125]. In contrast, inhibition of adrenergic neural activity enhances oxidative phosphorylation in endothelial cells through increased cytochrome c oxidase assembly factor 6 (COA6) expression, thereby inhibiting angiogenesis [125].

## Effects of chronic stress on the perineural nerve of tumor

Growing pre-clinical and clinical evidence suggests sustained adrenergic signaling in the TME plays a critical role in tumor growth and progression [90, 126]. However, the exact mechanism by which adrenergic neurotransmitters are delivered to the TME is unclear. Neurotrophic factors in the TME promote axonal growth of preexisting nerves. Thereby tumor-associated neural networks are established, which generate neural signals to regulate tumorigenesis and metastasis [127, 128].

Data to date suggest that peripheral nerves are present in a wide range of cancers and influence cancer behavior, closely related to tumorigenesis, angiogenesis, invasion, and metastasis. Patients with densely innervated tumors have increased metastasis rates and decreased survival compared to patients with less innervated tumors [125, 127, 129]. The neuronal organization in tumors has two origins. One is that tumor cells secrete neurotrophic factors that promote the production of new tumor-guided axons from pre-existing local nerves [130–133]. The other is the transformation of tumor cells into a neuroendocrine phenotype that recruit of neural progenitor cells from the CNS to infiltrate and reside in the TME and metastases [134, 135].

**Fig. 4:**
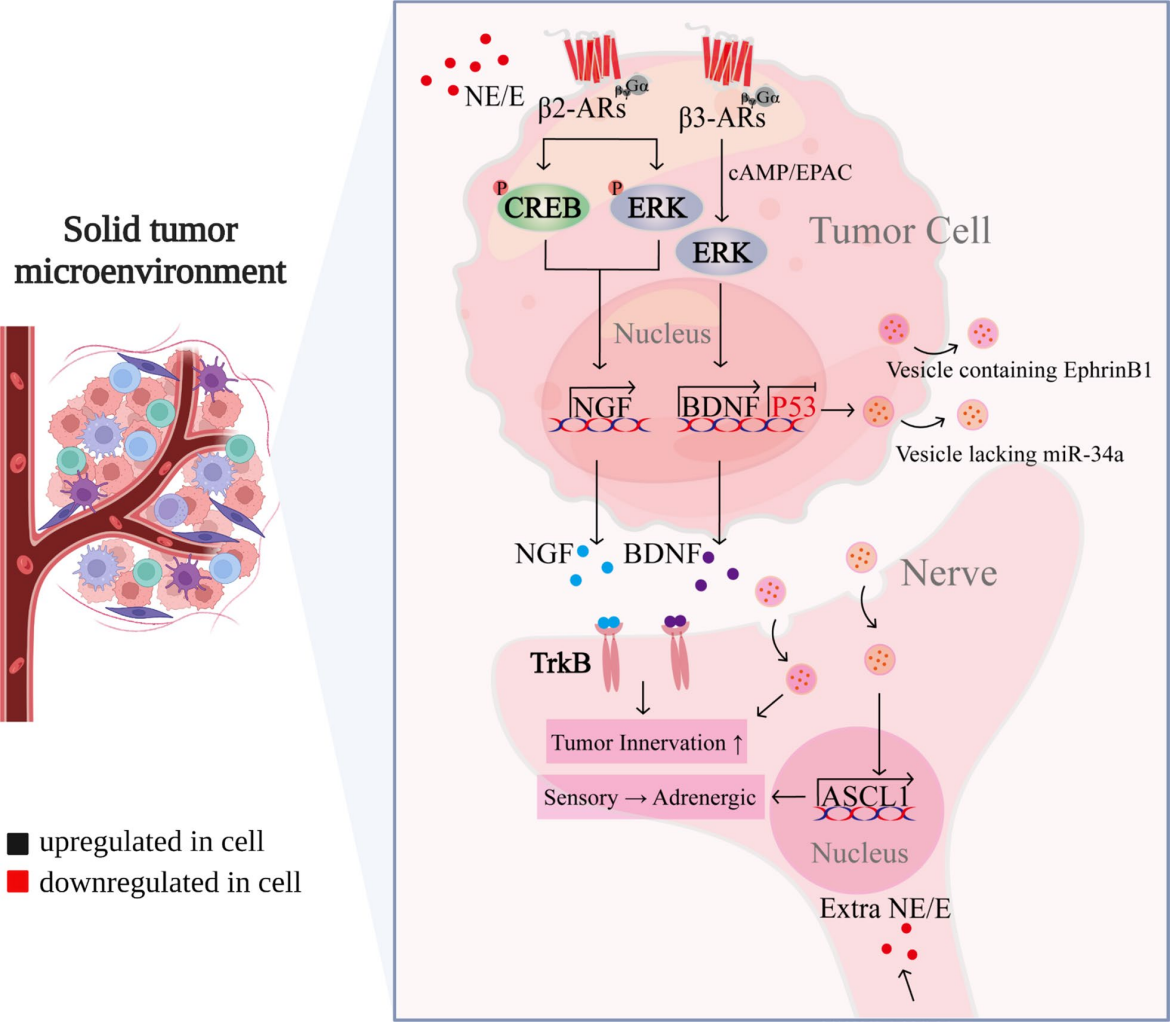
The biological mechanism of chronic stress affecting perineural nerve of tumor. Chronic stress-induced ADRB increases BDNF via ADRB3/cAMP/EpAC/JNK and NGF via ADRB2/CREB or ADRB2/ERK. BDNF and NGF bind to TrkB in peritumor sensory neurons, promoting innervation of the tumor microenvironment and producing a sustained accumulation of adrenergic signals, forming a feed-forward loop. Chronic stress-induced low p53 in tumor cells leads to miR-34a loss in exosomes, resulting in increased ASCL1 in peritumor sensory neurons, which redifferentiates peritumor sensory neurons into adrenergic neurons. In addition, tumor cells secrete exosomes containing EphrinB1 into peritumor sensory nerves, inducing tumor innervation

Allen et al*.* find that norepinephrine causes tumor cells to secrete brain-derived neurotrophic factor (BDNF) in an β3-ARs/cAMP/EPAC/JNK-dependent manner [130]. Similarly, in pancreatic ductal adenocarcinoma, norepinephrine promotes β2-ARs-dependent nerve growth factor (NGF) secretion [131]. Elevated BDNF/NGF levels promote innervation via neurotrophic receptor tyrosine kinase 2 (TrkB) receptors [130, 136], and overexpression of TrkB is related to shorter survival with ovarian cancer patients (Fig. 4) [137]. Interestingly, neurotrophic factors can also be released into the TME in the form of exosomes. As pointed out by Madeo, human head and neck squamous carcinoma cells can induce tumor innervation by secreting exosomes containing neurotrophic factor Ephrin B1 to peripheral sensory nerves (Fig. 4) [133]. Tumor innervation further increases adrenergic input into the TME, forming a feed-forward loop [131, 136]. Sustained accumulation of adrenergic signaling promotes tumor progression [130].

The sustained accumulation of adrenergic signaling can act not only through nerve growth factor (BDNF, NGF) but also by reprogramming sensory neurons in cancerous tissue to differentiate into new adrenergic neurons. Amit et al*.* show that in tumorigenesis, TP53 mutations in oral cancer cells leads to miR-34a loss in exosomes, which increases the expression of adrenergic-related mRNAs (e.g. ASCL1) in sensory neurons to promote differentiation of the adrenergic phenotype (Fig. 4). Then adrenergic neurons release local norepinephrine or epinephrine into the TME, resulting in tumor nerve density increased and tumor growth, providing an additional source of catecholaminergic in chronic stress models [132].

Under stressful conditions, CNS activates the autonomic nervous system or the HPA axis, secreting variety of mediators that are beneficial to tumorigenesis and progression. Moreover, CNS also directly establish contact with the distant TME to regulate tumor development. Mauffrey et al. found that neuronal progenitors located in the CNS express dual corticotropin, which is able to infiltrate and reside in prostate tumor tissues as well as their metastases. These neurons are able to differentiate into adrenergic type that contributed to the growth and metastasis of the primary tumor [135]. These data indicate the potential mechanisms by which stress hormones enter the TME, activate β-ARs pathways to promotes tumor development. However, whether neuronal infiltration into tumor tissues and reprogramming of neurons innervating tumor tissues to an adrenergic phenotype are specific phenomena for some cancers requires further investigation.

Increased tumor innervation in turn regulates angiogenesis, immune and inflammatory and oncogene activation through adrenergic signaling, promoting tumor growth and metastasis [138]. Not only that, tumor-associated neurons release the calcitonin gene-related peptide (CGRP), which directly increases the exhaustion of CD8^+^ T-cells and limits their ability to eliminate melanoma [139]. In contrast, sensory neuron ablation reverses these phenomena [140]. The data suggest that tumor nerve infiltration represents a new feature of cancer. And interfering with nerve signaling in TME through surgical or pharmacological approaches offers a promising new strategy for cancer treatment.

## Treatment for cancer patients with chronic stress

Epidemiological studies have shown that depression is related to cancer recurrence and mortality and it plays a key role as an independent predictor of cancer recurrence and prognosis [42, 141]. Obviously, the stress perception of cancer patients is affected by the physical and psychological burden of the disease. In recent years, based on studies in animal models, the endocrine, cellular and molecular mechanisms by which stress can promote cancer progression have been elucidated, including direct effects on the malignancy of cancer cells, anti-tumor immune activity, and indirect regulation of peritumor nerves. However, in clinic, there is lack of effective treatment for chronic stress in cancer patients [13]. We will discuss the opportunities and challenges of potential pharmacological and non-pharmacological treatment based on preliminary clinical data (Table 1) and retrospective analysis (Table 2).

**Table 1:** Clinical studies on BBs

Cancer	Sample size	Phase	Status	Outcome	β-Blocker subtype used in the study	Assessed perioperative β-blocker use	Combination of drugs	References
BC	1144	3	Completed	Improving PFS with advanced HER2-negative BC patients	Bisoprolol, metoprolol, atenolol, propranolol	No	Ramucirumab (IMC-1121B) docetaxel	NCT00703326
BC	2	2	Completed	Zero AEs have been reported/observed during and at the end of treatment	Propranolol	No	No	NCT02596867
BC	262	3	Completed	Prevention of LVEF decline and cardiac remodeling related to cancer treatment	Bisoprolol	No	Ramipril	NCT02236806
BC	82	2	Completed	Not reported	Carvedilol	No	Herceptin	NCT02177175
BC	20	1	Completed	The use of standard-of-care treatments for left ventricular impairment using ACE-I and BBs for trastuzumab therapy-induced left ventricular dysfunction is safe and improves LVEFs	Carvedilol	No	Ramipril	NCT02907021
BC	130	2	Completed	Early adjuvant treatment of anthracycline-containing is associated with a decrease in LVEF during extended follow-up	Metoprolol	No	Candesartan	NCT01434134
BC	450	2	Ongoing	Not reported	Carvedilol	No	No	NCT03879629
BC	100	2	Ongoing	Not reported	Bisoprolol	No	Perindopril	NCT04588935
BC	32	Not applicable	Completed	Decreased pro-metastatic and invasive markers	Propranolol	Yes	Etodolac	NCT00502684
BC	200	3	Completed	Using carvedilol reduces troponin levels and diastolic dysfunction	Carvedilol	No	Anthracycline (ANT) chemotherapy	NCT01724450
BC (locally advanced malignant neoplasm)	10	2	Completed	One AEs have been reported/observed during and at the end of treatment	Propranolol	No	Paclitaxel or Doxorubicin and Cyclophosphamide	NCT01847001
Metastatic HER-2-positive BC	817	3	Ongoing	Not reported	Carvedilol	No	No	NCT03418961
HER2 positive BC	150	2	Ongoing	Not reported	Carvedilol	No	No	NCT02993198
HER2 positive BC	31	2	Completed	Minimizing the risk of poor cardiac outcomes	Carvedilol	No	Herceptin or Perjeta or Kadcyla	NCT01904903
Hemangioma	6	1	Completed	Hemangioma size improved in 75% of subjects	Timolol	No	No	NCT01147601
Infantile hemangiomas	377	3	Completed	Compare with propranolol, atenolol has similar efficacy and fewer adverse events	Propranolol, atenolol	No	No	NCT02342275
Infantile hemangiomas	512	2, 3	Completed	Propranolol is effective in the treatment of infantile hemangioma	Propranolol	No	No	NCT01056341
Adults with spinal hemangioma	1000	4	Ongoing	Not Reported	Atenolol, propranolol	No	No	NCT05106179
Multiple myeloma	25	2	Completed	It is feasible to recruit and treat multiple myeloma patients with propranolol during Hematopoietic cell transplant (HCT)	Propranolol	No	No	NCT02420223
Multiple myeloma	90	3	Completed	Enalapril and carvedilol combination therapy prevent chemotherapy-induced left ventricular systolic dysfunction (LVSD) in patients with malignant hemopathies treated	Carvedilol	No	Enalapril	NCT01110824
Colorectal neoplasms	200	2	Ongoing	Perioperative inhibition of β-ARs and COX-2 provides a reliable and effective strategy for inhibiting multiple pathways associated with metastasis and cancer recurrence	Propranolol	Yes	Etodolac	NCT03919461
Colorectal neoplasms	400	3	Completed	No association between post-diagnostic BB use and CRC-specific mortality	Propranolol	No	Etodolac	NCT00888797
Ovarian, primary peritoneal, or fallopian tube cancer	32	1	Completed	Not reported	Propranolol hydrochloride	No	No	NCT01504126
Invasive epithelial ovarian cancer, primary peritoneal carcinoma, fallopian tube cancer	24	1	Completed	Not reported	Propranolol	No	No	NCT01308944
Skin melanoma	450	2	Suspended	BBs are associate with lower risk of melanoma recurrence and mortality	Propranolol hydrochloride	No	No	NCT01988831
Stage IIIC cutaneous melanoma AJCC v7, stage IV cutaneous melanoma AJCC v6, and v7	47	1,2	Ongoing	Not reported	Propranolol hydrochloride	No	Pembrolizumab	NCT03384836
Pancreatic neoplasms	210	2	Ongoing	Numerous perioperative pro-metastatic markers are reduced in blood and resected tumors	Propranolol	Yes	Etodolac	NCT03838029
Plasma cell myeloma	220	Not applicable	Ongoing	Not reported	Propranolol	No	No	NCT05312255
Esophagel adenocarcinoma	60	2	Ongoing	Not reported	Propranolol	No	Carboplatin and radiation therapy	NCT04682158
Non-small cell lung cancer, adenocarcinoma	345	3	Completed	PFS has been improved in lung cancer patients, delaying resistance to EGFR TKI	Not reported	No	Cisplatin, pemetrexed, afatinib	NCT00949650
Bladder cancer	242	2	Ongoing	Not reported	Propranolol hydrochloride	No	BCG	NCT04493489
Advanced cancer (cachexia)	15	Not applicable	Completed	Cancer patients who experience cachexia gain weight	Atenolol	No	Graded resistance training and aerobic exercise and melatonin and Juven and ibuprofen	NCT00625742

*AEs* adverse event, *BC* breast cancer, *LVEF* left ventricular ejection fraction, *PFS* progression-free survival

**Table 2:** Retrospective analysis of BBs

Cancer	Sample size	Phase	Outcome	β-Blocker subtype used in the study	Assessed perioperative β-blocker use	Combination of drugs
BC	610	Not applicable	Fail to corroborate BBs have a potential antitumor effect [152]	Not reported	No	No
BC	1413	Not applicable	Associate with improved RFS [167]	Not reported	No	Neoadjuvant chemotherapy (anthracyclines and taxanes)
BC	1413	Not applicable	Reduce distant metastasis, cancer recurrence, and cancer-specific mortality [143]	Propranolol	No	No
BC	18,733	Not applicable	Reduce the risk of BC recurrence is not supported [155]	Metoprolol, sotalol	No	No
TNBC	800	4	Associate with a lower risk of BC recurrence and death [142]	Carvedilol, sotalol, atenolol, betaxolol, bisoprolol, metoprolol, nebivolol	No	No
EOC	185	Not applicable	Perioperative BBs use is associated with longer OS among patients undergoing primary ovarian cancer debulking surgery [148]	Not reported	Yes	No
EOC	248	Not applicable	Associate with a 54% lower chance of death compared with patients not on BBs [169]	Not reported	No	Cytoreductive surgery and platinum-based chemotherapy
EOC	801	Not applicable	A selective beta-blocker (SBB) combination does not affect outcomes in patients receiving primary EOC therapy [160]	Not reported	No	No
EOC	1425	Not applicable	Nonselective BBs use is associated with longer OS [149]	Not reported	No	No
Stage II–IV EOC	32	Not applicable	Markers leading to adrenergic stress response have been reduced before surgery and during initial chemotherapy; BBs may improve baseline quality of life (TPO) and overall QOL, anxiety, and depression [172]	Propranolol	No	No
Invasive EOC	743	Not applicable	No clear relationship between BBs and ovarian cancer mortality [159]	Not reported	No	No
NSCLC	77	3	Postoperative radiotherapy and chemotherapy improve OS and DMFS [165]	Propranolol	No	Cisplatin and radiation therapy
NSCLC	435	1,2,3	Administration of BBs during the perioperative period did not improve RFS or OS in patients undergoing resection [157]	Not reported	Yes	No
NSCLC	722	Not applicable	Associated with improved locoregional PFS, DMFS, disease-free survival (DFS), and OS [168]	Not reported	No	Radiation therapy
NSCLC	1753	3	Not affect OS in stage III inoperable NSCLC [158]	Propranolol	No	No
Metastatic NSCLC	107	Not applicable	BBs use during CT may be associated with an improved OS for patients [147]	Metoprolol	No	No
Lung cancer	3340	Not applicable	Not associated with reduced mortality [156]	Not reported	No	No
Melanoma	741	Not applicable	Associated with a reduced risk of melanoma recurrence and death [145]	Not reported	No	No
Melanoma	53	Not applicable	BBs protect patients from disease recurrence [146]	Propranolol	No	No
Metastatic melanoma	9	1	The combination therapy is a safe prospective clinical trial with preliminary synergistic antitumor activity [166]	Propranolol	No	Pembrolizumab
Metastatic melanoma	195	Not applicable	A strategy of BBs combined with immunotherapy can improve OS [170]	Not reported	No	IL-2, αCTLA-4 and/or αPD-1 therapy
CRC	1975	4	BBs may be associated with longer survival in patients with stage IV CRC [144]	Not reported	No	No
Metastatic CRC	514	Not applicable	The combination of BBs and bevacizumab improved PFS and OS [171]	Not reported	No	Bevacizumab
Pancreatic and prostate cancer	3462	Not Applicable	Not supporting BBs improve survival in common cancers [153]	Not reported	No	No
Breast, colorectal, lung, or prostate cancer	15,582	Not applicable	BBs are not associated with improved survival in patients, and BBs did not affect survival in any cancer [154]	Not reported	No	No
PDAC	2054	Not applicable	May improve survival in PDAC patients [150]	Not reported	No	No
Clear-cell renal cell carcinoma (ccRCC)	913	Not applicable	Use of BBs within 90 days before surgery is not associated with PFS, CSS, or OS [161]	Not reported	No	No

*BC* breast cancer, *CRC* colorectal cancer, *DMFS* distant metastasis-free survival, *EOC* epithelial ovarian cancer, *NSCLC* non-small cell lung cancer, *OS* overall survival, *PDAC* pancreatic ductal adenocarcinoma, *PFS* progression-free survival, *RFS* relapse-free survival, *TNBC* triple-negative breast cancer

### Pharmacological treatment

#### Inhibiting tumor growth by regulating the nervous system

As mentioned above, chronic stress affects the occurrence and development of tumors mainly through ARs/GRs pathway. The β-adrenergic blockers, a class of neuromodulator drugs which blocks catecholamines from binding to β-ARs, have potential in improving life quality, anxiety, and depression in cancer patients. Moreover, it also reduces distant metastasis, recurrence of cancer, and cancer-specific mortality [142–150]. In clinical trials, BBs are also used as anti-hypertensive agents to prevent chemotherapy-induced cardiac injury [151]. However, part retrospective analyses of BBs show (Table 2) that there are conflicting claims about whether BBs alone can improve OS and progression-free survival (PFS) in patients [152–161]. The potential benefits of BBs may be offset by anxiety and depression [159]. Notably, β-ARs have different roles across tumor types and there is selective heterogeneity in the BBs [160]. It is also possible that the impact of GRs is being ignored [7]. There are fewer clinical trials and retrospective analyses supporting the use of GRs antagonists alone to improve patient survival. To be sure, mifepristone cannot be as a single agent to treat breast cancer [162]. However, low-dose of mifepristone improves life quality and reduces tumor size in patients with uterine fibroids (NCT00133705) [163].

Major clinical challenges to the use of neuromodulator drugs may include lacking tumor specificity and potential side effects at effective doses for cancer treatment. Additional randomized controlled trials (RCTs) are required to ascertain whether these drugs will improve clinical outcomes of cancer.

#### Combating treatment resistance by disrupting stress adaptation

The key role of chronic stress in influencing clinical treatment is to enhance the radiation resistance and drug resistance of tumor cells through β-ARs, thereby blocking apoptosis. Ongoing clinical trials tend to combine BBs/GRs antagonists with radiotherapy/chemotherapy (Table 1) to counteract treatment resistance due to chronic stress. A completed clinical trial shows that BBs delay the resistance of tumor cells to EGFR tyrosine kinase inhibitors, and PFS is improved in lung cancer patients [164]. Retrospective analyses have shown (Table 2) that the combination of BBs with anti-angiogenic drugs, immunotherapy, radiotherapy, or chemotherapy prolongs overall patient survival [165–172], which provides a direction for anti-cancer treatment resistance.

#### Combined with immunotherapy to enhance anti-tumor immunity

Preclinical studies show that psychological stress regulates the immune microenvironment of tumors by remodeling immune cells in the body. New evidence suggests that BBs may enhance the efficacy of cytotoxic therapies by modulating immune responses [92]. Retrospective analysis shows that combining immunotherapy with BBs improves the OS of patients with metastatic melanoma (Table 2) [166, 170]. BBs also prevent immune escape induced by chronic stress and improve survival in pancreatic cancer [173]. This finding may have important implications for immune checkpoint inhibitor-silenced tumors (e.g., pancreatic cancer, prostate cancer) [174]. Therefore, the combination therapy of BBs and immune checkpoint for these tumors deserves further investigation. Given that many neurotropic drugs are already used for other indications, there may be some promise in combining these drugs with cytotoxic therapies, immune checkpoint blockade, or cancer vaccines.

### Non-pharmacological treatment

Many complementary and integrative therapies are used to reduce stress and improve life quality of cancer patients. Complementary and alternative medicine bring some benefits to patients beyond surgery and medications by improving life quality, boosting the immune system, and relieving symptoms caused by conventional therapies [175].

#### Psychological interventions

Recent findings support that multiple psychological interventions may be effective in relieving stress and strengthening social support [176, 177]. Mindfulness interventions typically focus on fostering greater self-awareness for reducing responsiveness under stress. Mindfulness interventions help cancer patients relieve the anxiety associated with cancer diagnosis, treatment and fear of recurrence, and provide psychological support [178]. A 6-week mindfulness-based stress reduction (MBSR) program, which includes meditation, relaxation, yoga, and daily practices, shows that participation in MBSR programs improves life quality and reduces stress and mood disorders in breast cancer patients [179]. This is manifested by reduced levels of cortisol and proinflammatory cytokines and the recovery of NK cell activity [180, 181]. Randomized trials also show that MBSR improves cancer patients’ fatigue, insomnia, life quality, and biological markers of health [182, 183]. However, most studies focus on breast cancer patients, so the success of psychological interventions in other types of cancer may not be generalizable.

#### Exercise

Exercise interventions during and after treatment have great potential to improve survival and reduce recurrence in cancer patients [184]. The potential effects of adding exercise to conventional treatment to improve psychosocial, cognitive function, and depressive symptoms in cancer patients is being explored recently. Several RCTs have shown that physical activity during cancer treatment results in improved fatigue in cancer patients [185–187]. Exercise also improves treatment-related inflammatory markers levels in cancer patients [188], increases the CD8^+^ T cells activity [189], and promotes favorable immune environment restoration. Yoga is a form of aerobic exercise which is commonly used in mind–body therapy for breast cancer patients [190, 191]. In an RCT of breast cancer survivors, yoga reduces fatigue and inflammation and improves mood and life quality [192].

Taken together, physical exercise help to improve psychological stress, fatigue, and depression in cancer patients. We believe that trials with larger samples and longer follow-up periods should be conducted to assess the effects of exercise in patients with different malignancies and to determine whether this translates into a survival advantage.

#### Dietary strategies, herbs and nutritional products

Epidemiological data support the idea that dietary strategies have the potential to influence biological pathways associated with carcinogenesis [193]. Following a traditional dietary pattern, such as the Mediterranean, Norwegian or Japanese diets, and increasing the intake of fruits, vegetables, whole grains, nuts and seeds have anti-cancer and anti-depressive effects [194, 195]. RCTs illustrate that low-calorie diet combines with exercise promote the mental and physical health of depressed patients [196, 197].

The most frequently used complementary and alternative medicine are vitamins and herbal products [198]. Vitamin and mineral supplementation are beneficial to improve stress, anxiety and fatigue. A prospective study of breast cancer indicates that *Withania somnifera* improve cancer-related fatigue and life quality [199]. *Withania somnifera* is an herb that prevents oxidation, inflammation, cancer and stress.

However, vitamins, minerals and herbs may have a negative impact on treatment. A recent ancillary study shows that any anti-oxidant (vitamins A, C and E, coenzyme Q10) during treatment is correlated with an increased risk of recurrence and mortality [200]. Iron and vitamin B12 supplementation before and during chemotherapy are associated with an increased risk of death [200]. Given the present data, although the intake of natural products may relieve cancer-related stress and improve life quality, vitamins or minerals, especially during chemotherapy, ought to be used with caution by patients and clinicians.

## Conclusions and perspectives

There is strong evidence that chronic stress contributes to cancer development by modulating most features of cancer. Molecular and systemic mechanisms mediating these effects have been identified. Chronic stress affects the occurrence of various important events that promote cancer progression through ARs/GRs, including maintenance of stem cell-like traits, metastasis, angiogenesis, DNA damage accumulation, apoptosis resistance, immune escape, and metabolic reprogramming. These findings provide a basis for treating stress-related tumors. Stress signaling reprograms immune cells through glycometabolism, disrupts immune checkpoints, and regulates the distribution of immune cells in TME. Understanding the mechanisms by which chronic stress drives its immunosuppressive function may improve cancer immunotherapy, which could also be a potential breakthrough point for the current immunotherapy lack of effectiveness and help to discover new therapeutic approaches. Chronic stress also affects tumor-associated stromal cells to reshape TME. In addition, chronic stress affects tumor neural infiltration, which provides a potential mechanism for how stress hormones enter the TME. This reminds us to focus on the impact of chronic stress in cancer treatment, and timely take psychological intervention to relieve the chronic stress of patients.

Epidemiological and clinical studies have shown that stress affects cancer recurrence and the effectiveness of cancer treatments. And the use of synthetic glucocorticoids as anti-inflammatory and analgesic adjuvant drugs in cancer treatment affects the effectiveness of immunotherapy, which is also currently one of the problems that need to be solved in clinical practice. By modulating the nervous system and disrupting stress adaptation mechanisms, the use of BBs in synergy with anti-angiogenic drugs, immunotherapy, radiotherapy, or chemotherapy might be effective in treatment, but RCTs are needed to examine this hypothesis. Meanwhile, complementary and alternative medicine helps cancer patients improve their life quality from various aspects of psychological interventions, exercise, dietary strategies, herbs and nutritional additives and herbs and nutritional additives, relieving cancer patients' anxiety, then alleviating tumor progression. These pharmacological and non-pharmacological approaches against chronic stress provide novel interventional treatments for cancer.

## Data Availability

Not applicable.

## Abbreviations

ACTH: Adrenocorticotropic hormone
ARs: Adrenergic receptors
BBs: β-Adrenergic blockers
BDNF: Brain-derived neurotrophic factor
CAFs: Cancer-associated fibroblasts
CCL2: C-C motif chemokine ligand 2
CCR2: C-C motif chemokine receptor 2
CDK: Cyclin-dependent kinase
CRH: Corticotropin-releasing hormone
CNS: Central nervous system
COX-2: Cyclooxygenase2
CSCs: Cancer stem cells
DCs: Dendritic cells
Dex: Dexamethasone
EGFR: Epidermal growth factor receptor
FAK: Focal adhesion kinase
FAO: Fatty acid oxidation
GILZ: Glucocorticoid-induced leucine zipper
GLUT1: Glucose transporter protein 1
GRs: Glucocorticoid receptors
HDAC2: Histone deacetylase 2
hTERT: Human telomerase reverse transcriptase
HPA: Hypothalamic–pituitary–adrenal
LDHA: Lactate dehydrogenase A
MACC1: Metastasis‐associated in colon cancer 1
MBSR: Mindfulness-based stress reduction
MDSCs: Myeloid-derived suppressor cells
MMP: Matrix metalloproteinase
NGF: Nerve growth factor
NSCLC: Non-small cell lung cancer
OS: Overall survival
PFC: Prefrontal cortex
PFS: Progression-free survival
PGE2: Prostaglandin E2
PPARγ: Peroxisome proliferator-activated receptor γ
RCTs: Randomized controlled trial
ROR1: Receptor-tyrosine-kinase-like orphan receptor
SGK1: Serum glucocorticoid-induced kinase 1
SNS: Sympathetic nervous system
SYP: Synaptophysin
TAMs: Tumor-associated macrophages
TEAD4: TEA domain transcription factor 4
TILs: Tumor-infiltrating lymphocytes
TME: Tumor microenvironment
TrkB: Tyrosine kinase 2
USP28: Ubiquitin-specific protease 28
VEGF: Vascular endothelial growth factor
